# Genome-wide variations analysis of sorghum cultivar Hongyingzi for brewing Moutai liquor

**DOI:** 10.1186/s41065-020-00130-4

**Published:** 2020-05-14

**Authors:** Lingbo Zhou, Can Wang, Xu Gao, Yanqing Ding, Bin Cheng, Guobing Zhang, Ning Cao, Yan Xu, Mingbo Shao, Liyi Zhang

**Affiliations:** Institute of Upland Food Crops, Guizhou Academy of Agricultural Sciences, Guiyang, 550006 Guizhou China

**Keywords:** Sorghum, Hongyingzi, Genome-wide variations analysis, Whole-genome resequencing technology

## Abstract

**Background:**

Hongyingzi is a sorghum (*Sorghum bicolor* L. Moench) cultivar for brewing Moutai liquor. For an overall understanding of the whole genome of Hongyingzi, we performed whole-genome resequencing technology to reveal its comprehensive variations.

**Results:**

Compared with the BTx623 reference genome, we uncovered 1,885,774 single nucleotide polymorphisms (SNPs), 309,381 small fragments insertions and deletions (Indels), 31,966 structural variations (SVs), and 217,273 copy number variations (CNVs). These alterations conferred 29,614 gene variations. It was also predicted that 35 gene variations were related to the multidrug and toxic efflux (MATE) transporter, chalcone synthase (CHS), ATPase isoform 10 (AHA10) transporter, dihydroflavonol-4-reductase (DFR), the laccase 15 (LAC15), flavonol 3′-hydroxylase (F3′H), flavanone 3-hydroxylase (F3H), *O*-methyltransferase (OMT), flavonoid 3′5′ hydroxylase (F3′5′H), UDP-glucose:sterol-glucosyltransferase (SGT), flavonol synthase (FLS), and chalcone isomerase (CHI) involved in the tannin synthesis.

**Conclusions:**

These results would provide theoretical supports for the molecular markers developments and gene function studies related to the tannin synthesis, and the genetic improvement of liquor-making sorghum based on the genome editing technology.

## Introduction

Sorghum [*Sorghum bicolor* (L.) Moench] is the fifth largest cereal crop in the world after corn (*Zea mays* L.), wheat (*Triticum aestivum* L.), rice (*Oryza sativa* L.), and barley (*Hordeum vulgare* L.), which is widely distributed in the arid and semi-arid regions of the tropics, and also one of the earliest cultivated cereal crops in China [1]. It has become a model crop for genome research of cereal crops because of its wide adaptability to environment, strong stress resistance, rich resources, and relatively small genome [2, 3]. According to different purposes, sorghum are generally divided into three types, namely sweet sorghum, feed sorghum, and grain sorghum [4]. Sorghum is one of the main raw materials for Moutai-flavor liquor and Luzhou-flavor liquor production due to its high amylopectin content and tannin content [5, 6]. In recent years, the undiversified main liquor-making sorghum cultivar and its continuous degradation phenomenon has affected the supply of raw materials for liquor-making sorghum and restricted the development of liquor enterprises [7]. Therefore, investigation of liquor-making sorghum genetic resources is a crucial measure for better straight evolution, genetic studies, and liquor-making sorghum breeding strategies.

Genetic variation is a kind of variation that can be passed on to offspring due to the changes of genetic material in organisms and leads to the genetic diversity at different levels. There are many types of genetic variation in the genome, from microscopic chromosome inversion to single nucleotide mutation. With the development of genomics, the information of genetic variation that can be studied has become more comprehensive, such as single nucleotide polymorphism (SNP), small fragments insertion and deletion (Indel), structural variation (SV), and copy number variation (CNV) [8–10]. With the rapid development of molecular biology, whole-genome resequencing technology has been applied to genome-wide variations analysis in *Arabidopsis*, rice, maize, tomato, and other plants [8, 11–13]. The whole genome sequences of grain sorghum cultivar BTx623 has provided a template for genome-wide variations analysis in sorghum [14], and the first genome-wide variations analysis of sorghum was reported by [15].

Tannin, also known as condensed tannins or proanthocyanidins, is oligomer and polymer of flavan-3-ols. It is widespread throughout the plant kingdom, with diverse biological and biochemical functions, such as protection against predation from herbivorous animals and pathogenic attack from bacteria and fungi [16]. Tannin is found in seeds of sorghum with a pigmented testa layer and it has been shown high antioxidant and dietary fiber levels, and to decrease protein digestibility and feed efficiency in humans and animals [17]. High tannin content is the main reason why sorghum has become a raw material for brewing famous liquor and tannin content is closely related to the brewing flavor, such as sorghum cultivar with between 1 and 2% of tannin content is raw material for brewing Moutai-flavor liquor [18, 19]. Previous studies have mapped some gene loci associated with tannin content of sorghum. The *Tan1* gene (*Sb04g031730*) was cloned, which code a WD40 protein and control the tannin biosynthesis [16]. Two gene loci linked to tannin content were also found [20]. One was named as *Sb01g001230*, coding glutathione-S-transferase, and another was named as *Sb02g006390*, coding bHLH transcription factor and was isotopic with gene *B*_*2*_ for color seed coat.

Hongyingzi, a sorghum cultivar used for brewing Moutai liquor containing 83.40% total starch, 96.27% amylopectin/total starch ratio, and 1.61% tannin. However, the genome information of Hongyingzi is not fully understood. Here, we used whole-genome resequencing technology to resequence Hongyingzi genome to identify patterns of sequence polymorphism and structural variation in comparison with the published BTx623 genome. This effort identified a large quantity of SNPs, Indels, SVs, and CNVs. Comparison of these variation data defined potential genome regions and metabolic pathways associated with tannin synthesis. Such knowledge are useful for genetic improvement and tailor-designed breeding of liquor-making sorghum.

## Materials and methods

### Plant materials

Hongyingzi was used in this study, which approved by the Guizhou Crop Cultivar Approval Committee (Guiyang, Guizhou Province, China) in 2008, is a medium maturity sorghum cultivar used for brewing Moutai liquor and developed by Renhuai Fengyuan Organic Sorghum Breeding Center at Guizhou, China in 2008 [21]. Seeds of Hongyingzi were sterilized by soaking in 0.1% mercury dichloride for 15 min, and then rinsed with distilled water for ten times. Next, seeds were placed in a germination box lined with three layers of filter paper and added 15 mL distilled water. The germination box was placed in the RXZ-1000B artificial climate box for cultivating 10 days as following parameters settings, day/night temperature is 28 °C/25 °C, light/dark time is 12 h/12 h, humidity is 85%, and light intensity is 340 μmol m^− 2^ s^− 1^.

### DNA isolation and resequencing

The 10-day-old healthy seedlings were harvested for DNA extraction using the CTAB buffer method [22]. The DNA purity was determined by 0.8% agarose gel 100 V electrophoresis for 40 min and DNA concentration was determined by Qubit® 2.0 fluorescent meter (Invitrogen, Carlsbad, USA). Following quality assessment, the genomic DNA was randomly broken into 350 bp fragments by Covaris ultrasonic crushing apparatus and DNA fragments were end repaired, added ployA tail, added sequencing connector, purification, and PCR amplification to complete the establishment of the library. The constructed library was used to paired-end PE150 sequencing on Illumina HiSeq 4000 sequencing platform by Beijing Novogene technology co., LTD (Beijing, China).

### Filtering reads and mapping reads

The original image data generated by the sequencing machine were converted into sequence data via base calling (Illumina pipeline CASAVA v1.8.2) and then subjected to quality control procedure to remove unusable reads according to following criteria: the reads contain the Illumina library construction adapters, the reads contain more than 10% unknown bases (N bases), and one end of the read contain more than 50% of low quality bases (sequencing quality value ≤5). After filtration, sequencing reads were aligned to the BTx623 reference genome using BWA v0.7.8 [23] with the parameters as ‘mem –t 4 –k 32 -M’. Subsequent processing, including duplicate removal was performed using SAMtools v0.1.19 [24] with the parameter as ‘rmdup’. The BTx623 reference genome sequences were downloaded from the https://phytozome.jgi.doe.gov/pz/portal.html#!info?alias=Org_SbicolorRio_er.

### SNP and Indel analyses

SNP and Indel detections were performed using SAMtools v0.1.19 with the parameters as ‘mpileup -m 2 -F 0.002 -d 1000’. Based on the filtering and mapping results, the clean data were assembled and estimated using a Bayesian model. The performance of SNP detection was based on the following criteria: average quality of the novel allele > 20, adjacent SNPs were separated by ≥5 bp, and at least four reads supported the genotype. For Indel detecting, mapped reads met the pair-end requirements and contained alignment gaps in one end. We mapped the paired-end reads to the reference sequence by allowing up to 50 bp gaps. Then, gaps supported by at least three non-redundant paired-end reads were extracted. A potential Indel was identified when the number of ungapped reads were < 2. The annotations of SNP and Indel were performed using ANNOVAR software [25], and the information on genes with SNPs and Indels were downloaded from the *Sorghum bicolor* Genome Database [26].

### SV and CNV analyses

SV and CNV detections were performed using BreakDancer v1.4.4 [27] and CNVnator v0.3 [28] with the parameter as ‘-call 100’, respectively. According to the principle of paired-end sequencing, one read of a paired-end should be aligned to the forward sequence, while the other read of a paired-end should be aligned to the reverse sequence. The distance between the two aligned positions should be in accordance with the insert size. Thus, the alignment of the two paired reads to the genome is regarded to be of normal direction and appropriate spanning. If the direction or spanning of the alignments of the two paired reads is different from expectation, the region might have a structural variation. The abnormal paired-end alignments were analyzed by clustering and compared with the types of SVs as previously defined. The SVs were detected in the same manner, with support from at least two abnormal paired-end read. After determination of SVs, the total number of CNVs was counted. The annotations of SV and CNV were performed using ANNOVAR software, and the information on genes with SVs and CNVs were also downloaded from the *Sorghum bicolor* Genome Database.

### Gene variation analysis

Using the BTx623 gene set as the reference, genes with non-synonymous SNPs and Indels in coding regions identified in the Hongyingzi were selected as the candidate gene set. These genes were aligned to the NCBI using Blast2go JavaScript. Gene Ontology (GO) numbers were downloaded from the *Sorghum bicolor* Genome Database and imported to the WEGO database [29] for clustering analysis. Genes that were involved in the tannin synthesis were selected and mapped to Kyoto Encyclopedia of Genes and Genomes (KEGG) [30] sorghum pathway data and were examined for whether they are enriched in particular pathways based on the hypergeometric distribution test. Fisher’s exact test was used to identify pathways significantly enriched (*P* < 0.1) with tannin synthesis associated genes.

## Results

### Genome-wide identification of genetic variations in Hongyingzi

**Fig. 1:**
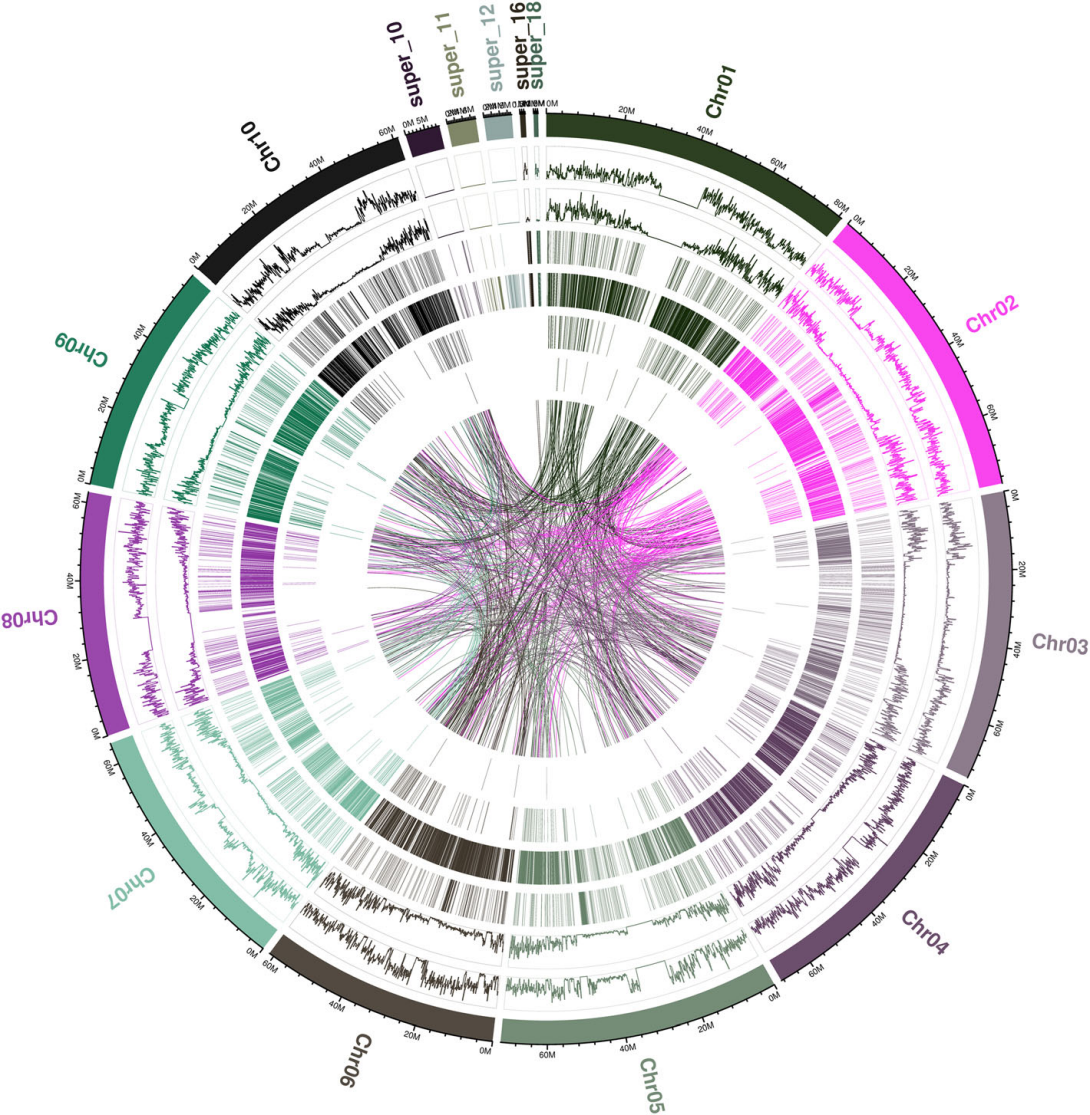
Genome-wide landscape of genetic variation in Hongyingzi. Cycles from outside to inside indicate chromosome, single nucleotide polymorphism (SNP), small fragments insertion and deletion (Indel), copy number variation (CNV) duplication and deletion, structural variation (SV) insertion, SV deletion, SV invertion, and SV intrachromosomal translocation (ITX) and SV interchromosomal translocation (CTX)

The whole genome of Hongyingzi was resequenced using Illumina Genome Analyser sequencing technology. The genome size of the BTx623 reference genome is 732.15 Mb. Resequencing yielded 45.84 Gb of raw data, which comprised 45.79 Gb of high quality clean data (Table 1). There was a high sequencing quality (Q20 ≥ 97.55%, Q30 ≥ 93.10) and the GC content was 44.30%. The results showed that the 297,504,853 reads obtained with Hongyingzi were mapped to the BTx623 reference genome, with an effective depth of 56.10 X coverage, 95.94% of coverage at least one base, and 94.17% of coverage at least four bases (Table 2). With these reads and the information from the BTx623 reference genome, large quantities of SNPs, Indels, SVs, and CNVs were identified (Fig. 1). Compared with the BTx623 reference genome, we finally found 1,885,774 SNPs, 309,381 Indels, 31,966 SVs, and 217,273 CNVs in Hongyingzi. These variations were distributed relatively evenly across 10 chromosomes of sorghum and had the most distribution on chromosome 1 as well as a few were distributed on extra chromosomes.

**Table 1:** Summary of resequencing data of Hongyingzi

Raw base (Gb)	Clean base (Gb)	Effect rate (%)	Error rate (%)	Q20 (%)	Q30 (%)	GC content (%)
45.84	45.79	99.82	0.03	97.55	93.10	44.30

**Table 2:** Sequence alignment of Hongyingzi to BTx623

Mapped reads	Total reads	Mapping rate (%)	Average depth (X)	Coverage at least 1 base (%)	Coverage at least 4 bases (%)
297,504,853	305,064,750	97.52	56.10	95.94	94.17

### SNPs in the Hongyingzi genome

A total of 1,885,774 SNPs were identified in the Hongyingzi genome, including 1,230,508 transitions and 655,266 transversions. Besides, there were 1,401,089 homozygous SNPs and 484,685 heterozygous SNPs (Fig. S1 in Additional file 1), and the het rate was 0.066%. As shown by annotations of SNPs detected in Hongyingzi (Table 3), there were 1,515,993 SNPs mutation in intergenic, 89,326 SNPs in 1 kb of upstream, 75,170 SNPs in 1 kb of downstream, and 6344 SNPs mutated in both 1 kb of upstream and another 1 kb of downstream. We found that 76,528 SNPs were mutated in exonic regions, including 38,176 synonymous SNPs, 37,774 non-synonymous SNPs, 453 SNPs related to gain of stop codons, and 125 SNPs related to loss of stop codons. We also found that there were 122,211 SNPs mutation in intronic regions and 202 SNPs in splicing sites. Besides, the proportion of C:G > T:A type was observed to be the highest (Fig. S2 in Additional file 1).

**Table 3:** Annotations of single nucleotide polymorphisms (SNPs), small fragments insertions and deletions (Indels), structural variations (SVs), and copy number variations (CNVs) detected in Hongyingzi

Category	Numbers of SNPs	Numbers of Indels	Numbers of SVs	Numbers of CNVs	Region
Intergenic	1,515,993	190,165	9661	17,082	
1 kb of upstream	89,326	38,198	1915	985	
1 kb of downstream	75,170	28,361	1460	789	
Upstream/downstream	6344	2779	176	96	
Gain of stop codons	453	103			Coding regions
Loss of stop codons	125	22			Coding regions
Synonymous	38,176				Coding regions
Non-synonymous	37,774				Coding regions
Frameshift (insertions)		1354			Coding regions
Frameshift (deletions)		1476			Coding regions
Non-frameshift (insertions)		3219			Coding regions
Non-frameshift (deletions)		3201			Coding regions
Exonic			3657	1822	
Intronic	122,211	40,223	1119	496	
Splicing sites	202	189	5		

### Indels in the Hongyingzi genome

A total of 309,381 Indels containing 149,071 insertions and 160,310 deletions, was uncovered in the Hongyingzi genome. These Indels also included 309,361 homozygous and 20 heterozygous Indels (Fig. S3 in Additional file 2), and the het rate was 0.0065%. Annotation analysis (Table 3) showed that there were 190,165, 38,198, 28,361, and 2779 Indels mutated in intergenic, 1 kb of upstream, 1 kb of downstream, and both 1 kb of upstream and another 1 kb of downstream, respectively. We found that 9375 Indels were mutated in exonic regions, in which 103 Indels were related to gain of stop codons, 22 Indels were related to loss of stop codons, 1354 insertions and 1476 deletions might lead to frameshift, and 3219 insertions and 3201 deletions might lead to non-frameshift. We also found 40,223 Indels were mutated in intronic regions and 189 Indels did in splicing sites. Besides, the proportion of 1 bp and 3 bp Indels (Fig. S4 in Additional file 2) were observed to be the highest in whole genome and coding regions, respectively.

### SVs in the Hongyingzi genome

A total of 31,966 SVs were identified in the Hongyingzi genome, including 70 insertions, 15,975 deletions, 1948 inversions, 4938 intrachromosomal translocations, and 9035 interchromosomal translocations (Fig. S5 in Additional file 3). As shown by annotations of SVs detected in Hongyingzi (Table 3), there were 9661 SVs mutation in intergenic, 1915 in 1 kb of upstream, 1460 in 1 kb of downstream, and 176 in both 1 kb of upstream and another 1 kb of downstream. We also found that there were 3657 SVs mutation in exonic regions, 1119 in intronic regions, and 5 in splicing sites.

### CNVs in the Hongyingzi genome

A total of 217,273 CNVs including 4966 duplications and 16,307 deletions was uncovered in the Hongyingzi genome (Fig. S6 in Additional file 3). Annotation analysis (Table 3) showed that there were 17,082, 985, 789, and 96 CNVs mutated in intergenic, 1 kb of upstream, 1 kb of downstream, and both 1 kb of upstream and another 1 kb of downstream, respectively. We also found that there were 1822 CNVs and 496 CNVs mutated in exonic and intronic regions, respectively.

### Functional clustering of gene variations

**Fig. 2:**
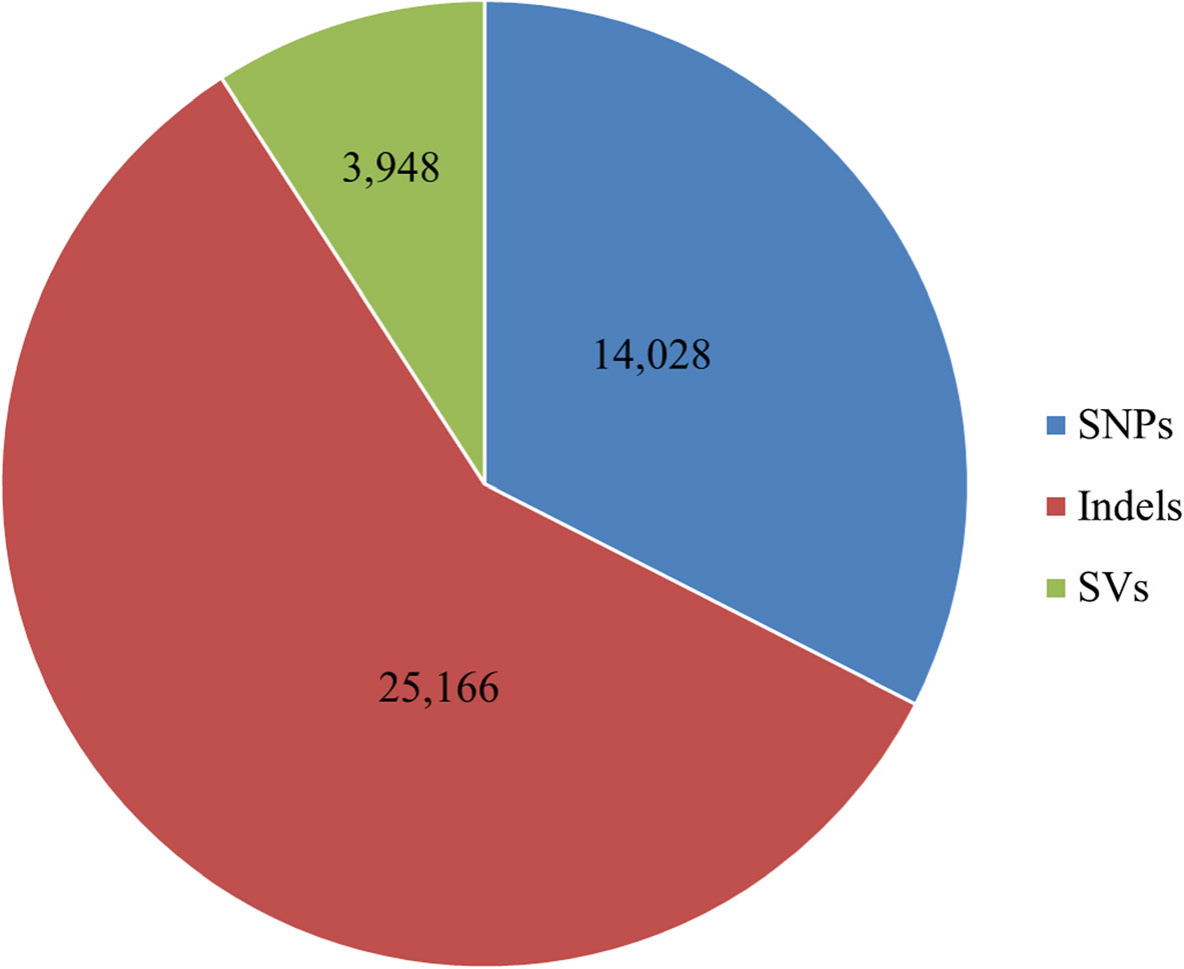
Summary of genes with three types of variations, single nucleotide polymorphism (SNPs), small fragments insertions and deletions (Indels), and structural variations (SVs). The blue pie indicates SNPs, the red pie indicates Indels, and the green pie indicates SVs

**Fig. 3:**
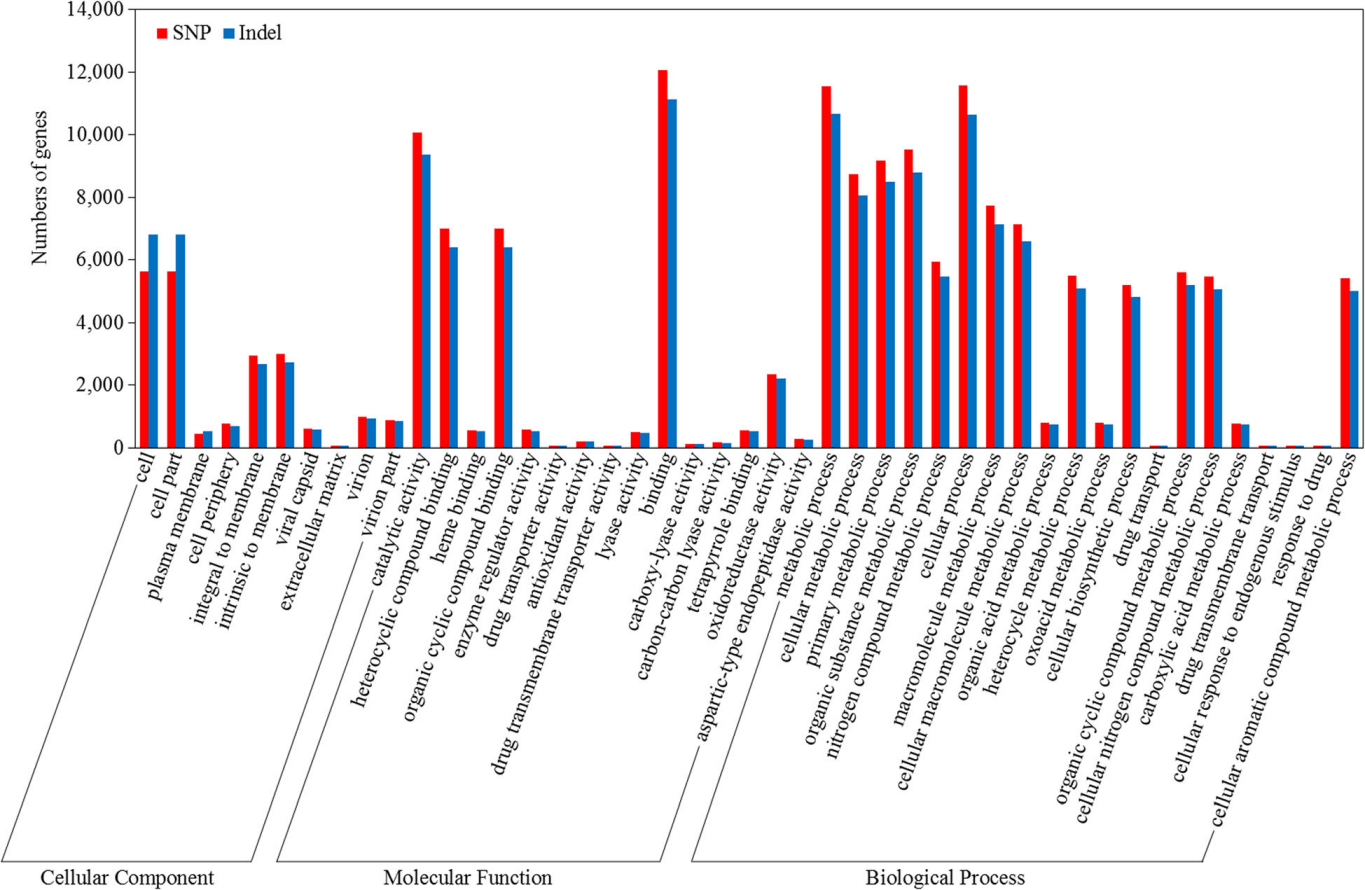
WEGO clustering of genes with three types of variations, single nucleotide polymorphism (SNPs) and small fragments insertions and deletions (Indels). The red bar indicates SNPs, the blue bar indicates Indels

**Fig. 4:**
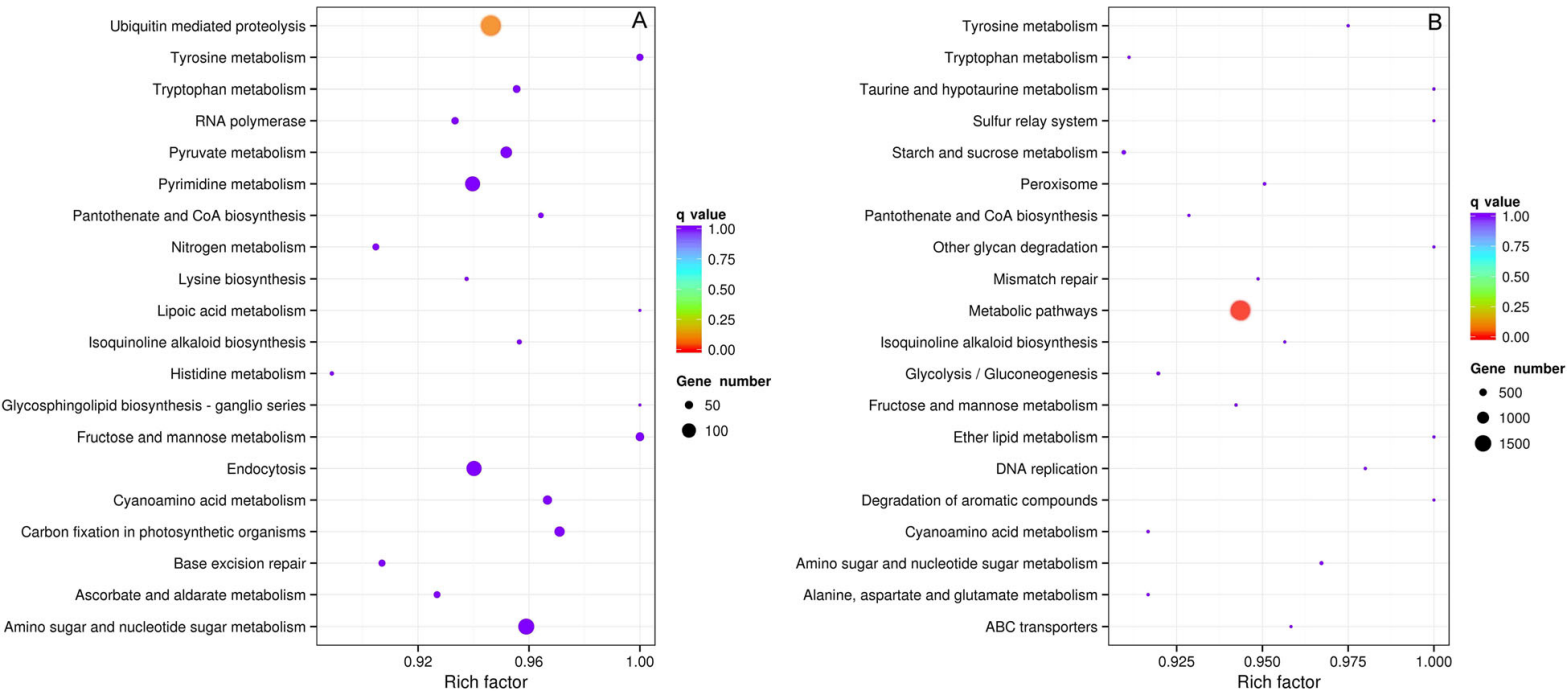
Classification of gene variations compared with Kyoto Encyclopedia of Genes and Genomes (KEGG) database. **a:** Top 20 significantly enriched pathways with single nucleotide polymorphism (SNPs) variations. **b**: Top 20 significantly enriched pathways with small fragments insertions and deletions (Indels) variations. Gene number: number of target genes in each term or pathway. Rich factor: the ratio of the number of target genes divided by the number of all the gene in each term or pathway

Compared to the BTx623 reference genome, 29,614 gene variations were identified in the Hongyingzi genome (Fig. 2). Of which, 14,028, 25,166 and 3948 was caused by SNPs, Indels, and SVs, respectively. GO annotation showed that SNPs and Indels were distributed among different gene ontologies (Fig. 3). In cellular component ontology, the cell and cell part contained the majority of gene variations with 19.06% SNPs and 23.01% Indels. Extracellular matrix contained a lower rate of variation. In molecular function ontology, binding and catalytic activity had a higher rate of variation. Binding included 40.77 and 37.56% of variation in SNPs and Indels, while catalytic activity did 34.01 and 31.67% of variation in SNPs and Indels. In biological process ontology, metabolic process and cellular process had a high rate of variation. Metabolic process term included 39.00 and 36.07% of variation in SNPs and Indels, while cellular process did 39.05 and 35.99% of variation in SNPs and Indels. In KEGG annotation, 141 gene variations caused by SNPs (Fig. 4a) involved in the ubiquitin mediated proteolysis, while 1756 caused by Indels (Fig. 4b) involved in the metabolic pathways. These variations may affect the distinguishing traits between Hongyingzi and BTx623.

### Gene variations involved in tannin synthesis

**Fig. 5:**
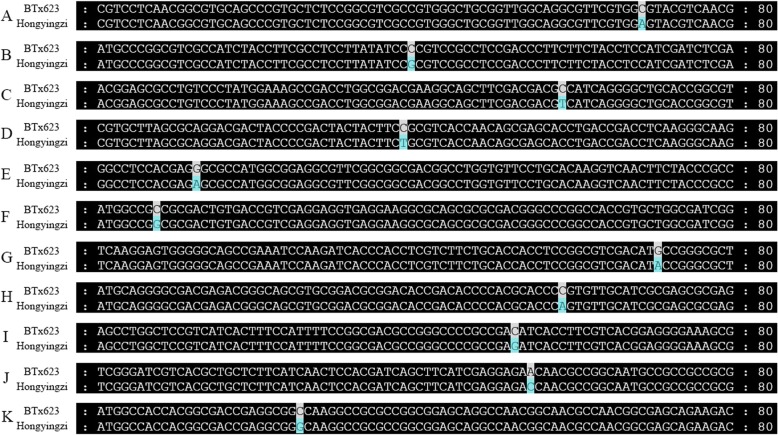
DNA sequence alignments of 11 genes that had only 1 bp non-synonymous SNP variation in coding regions. **a:***Sobic.001G012600*. **b:***Sobic.001G543900*. **c:***Sobic.003G230900*. **d:***Sobic.004G179000*. **e:***Sobic.004G310100*. **f:***Sobic.005G136200.***g:***Sobic.005G136300*. **h:***Sobic.007G165500*. **i:***Sobic.008G030100*. **j:***Sobic.010G207800*. **k**: *Sobic.010G052200*

Compared to the BTx623 reference genome, we found that 35 gene variations were related to the tannin synthesis in the Hongyingzi genome (Table 4). Of which, 7 genes did in the multidrug and toxic efflux (MATE) transporter, 7 involved in the chalcone synthase (CHS), 4 did in the ATPase isoform 10 (AHA10) transporter, 4 did in the dihydroflavonol-4-reductase (DFR), 3 did in the laccase 15 (LAC15), 2 did in the flavonol 3′-hydroxylase (F3′H), 2 did in the flavanone 3-hydroxylase (F3H), 2 did in the *O*-methyltransferase (OMT), 1 did in the flavonoid 3′5′ hydroxylase (F3′5′H), 1 did in the UDP-glucose:sterol-glucosyltransferase (SGT), 1 did in the flavonol synthase (FLS), and 1 did in the chalcone isomerase (CHI). We selected 11 genes with only 1 bp non-synonymous SNP variation in coding regions to DNA sequence alignment (Fig. 5), and found that these 11 genes altered the coding amino acids.

**Table 4:** Variation genes involved in tannin synthesis

Gene name	Chromosome	Annotation	Variation type	Variation information
*Sobic.001G012600*	1	*SbMATE*	Non-synonymous SNP	1,175,307 bp, C/A
*Sobic.001G185400*	1	*SbMATE*	Non-synonymous SNP	15,851,598 bp, G/A; 15,851,639 bp, C/T; 15,851,643 bp, G/C; 15,851,644 bp, G/C; 15,857,015 bp, G/A
			Non-frameshift insertion	15,851,633 bp, −/GGT
*Sobic.001G185500*	1	*SbMATE*	Non-frameshift insertion	15,867,230 bp, −/GCACGG
*Sobic.001G185600*	1	*SbMATE*	Non-synonymous SNP	15,877,514 bp, G/T; 158,77,530 bp, T/A; 15,879,898 bp, T/G
			Non-frameshift deletion	15,880,626 bp, ACCGGCGCC/−
*Sobic.004G349550*	4	*SbMATE*	Non-frameshift insertion	67,834,717 bp, −/GCTGCT
*Sobic.004G349600*	4	*SbMATE*	Non-synonymous SNP	67,848,132 bp, C/G; 67,848,135 bp, C/G; 67,848,250 bp, T/G
*Sobic.007G165500*	7	*SbMATE*	Non-synonymous SNP	60,025,990 bp, C/A
*Sobic.001G360800*	1	*SbF3′5′H*	Non-synonymous SNP	65,069,116 bp, T/C
*Sobic.001G543900*	1	*SbAHA10*	Non-synonymous SNP	80,740,375 bp, C/G
*Sobic.003G436400*	3	*SbAHA10*	Non-frameshift insertion	73,733,903 bp, −/CCG
*Sobic.010G063700*	10	*SbAHA10*	Non-synonymous SNP	5,033,142 bp, C/A; 5,033,614 bp, C/T; 5,033,877 bp, C/A; 5,034,044 bp, C/T; 5,034,053 bp, G/C
			Frameshift insertion	5,032,581 bp, −/GC; 5,032,758 bp, −/GAGC; 5,033,017 bp, −/ATCT
			Non-frameshift deletion	5,032,669 bp, GTGCTGTTC/−
			Non-frameshift insertion	5,033,742 bp, −/GGG; 5,034,105 bp, −/TTCCAC
			Gain of stop codons	5,034,223 bp, −/CTATTTCA
*Sobic.010G207800*	10	*SbAHA10*	Non-synonymous SNP	55,088,876 bp, A/C
*Sobic.002G117500*	2	*SbSGT*	Non-synonymous SNP	14,508,960 bp, C/A
*Sobic.002G310500*	2	*SbCHS*	Non-synonymous SNP	68,442,264 bp, A/C; 68,442,283 bp, G/A
*Sobic.004G179000*	4	*SbCHS*	Non-synonymous SNP	53,190,344 bp, C/T
*Sobic.005G135600*	5	*SbCHS*	Non-synonymous SNP	58,503,342 bp, T/A; 58,503,472 bp, T/C; 58,503,507 bp, G/A; 58,503,555 bp, C/G
*Sobic.005G136200*	5	*SbCHS*	Non-synonymous SNP	58,859,286 bp, C/G
*Sobic.005G136300*	5	*SbCHS*	Non-synonymous SNP	58,881,162 bp, G/A
*Sobic.005G137100*	5	*SbCHS*	Non-synonymous SNP	58,943,632 bp, C/T
*Sobic.008G036800*	8	*SbCHS*	Non-synonymous SNP	3,477,776 bp, G/A
			Non-frameshift deletion	3,477,795 bp, ACG/−
*Sobic.003G230900*	3	*SbDFR*	Non-synonymous SNP	57,029,960 bp, C/T
*Sobic.003G231000*	3	*SbDFR*	Non-frameshift deletion	57,041,941 bp, CTGGGA/−
*Sobic.004G050200*	4	*SbDFR*	Non-frameshift deletion	4,052,019 bp, AAC/−
*Sobic.009G043800*	9	*SbDFR*	Non-synonymous SNP	4,149,752 bp, T/C; 4,149,842 bp, G/A; 4,149,896 bp, G/T; 4,149,998 bp, T/C; 4,150,031 bp, C/G
*Sobic.004G200900*	4	*SbF3′H*	Non-synonymous SNP	55,234,140 bp, T/G
			Non-frameshift deletion	55,233,739 bp, CGGGAA/−
*Sobic.009G162500*	9	*SbF3′H*	Non-synonymous SNP	51,944,205 bp, A/G; 51,948,174 bp, C/G
*Sobic.004G236000*	4	*SbLAC15*	Non-synonymous SNP	58,382,355 bp, G/A; 58,382,419 bp, G/A; 58,383,602 bp, A/G; 28,383,682 bp, G/T
*Sobic.004G236100*	4	*SbLAC15*	Non-synonymous SNP	58,391,947 bp, C/T
			Frameshift deletion	58,392,294 bp, CTAC/−
*Sobic.005G156700*	5	*SbLAC15*	Non-synonymous SNP	62,814,031 bp, G/A; 62,814,043 bp, A/G; 62,814,250 bp. C/G
			Non-frameshift deletion	62,816,156 bp, CGTCAACGT/−
			Frameshift deletion	62,813,716 bp, C/−; 62,813,926 bp, A/−; 62,814,183 bp, C/−
			Frameshift insertion	62,813,832 bp, −/A; 62,814,474 bp, −/TA
*Sobic.004G310100*	4	*SbFLS*	Non-synonymous SNP	64,699,203 bp, G/A
*Sobic.006G253900*	6	*SbF3H*	Non-synonymous SNP	59,157,048 bp, A/T; 59,157,274 bp, C/T; 59,158,255 bp, T/A
*Sobic.006G254000*	6	*SbF3H*	Non-synonymous SNP	59,160,879 bp, A/C; 59,161,461 bp, G/A
*Sobic.007G047300*	7	*SbOMT*	Non-synonymous SNP	4,721,737 bp, G/C; 4,721,966 bp, C/T; 4,724,116 bp, T/C
*Sobic.010G052200*	10	*SbOMT*	Non-synonymous SNP	4,072,017 bp, C/G
*Sobic.008G030100*	8	*SbCHI*	Non-synonymous SNP	2,684,008 bp, C/G

## Discussion

The rapid development of high-throughput sequencing technologies and bioinformatic tools makes it possible to understand the genetic variation and diversity of sorghum at the whole genome level, and then are useful for genetic improvement and tailor-designed breeding of sorghum [14, 15, 31]. In this study, we used whole-genome resequencing technology to analyze the genetic variation in Hongyingzi, which is a sorghum cultivar for brewing Moutai liquor. The results showed that a lot of genome sequences were different between Hongyingzi and BTx623, and more than 2 million SNPs and Indels, along with large numbers of SVs and CNVs were identified. This is the first report on the genome-wide variations analysis in liquor-making sorghum, which will be valuable for further genotype-phenotype studies and for molecular marker assisted breeding of liquor-making sorghum.

In this study, the proportion of SNPs identified as in intronic regions was 6.48%. Compared to *Arabidopsis* [32], the intronic regions of sorghum genes harbor more SNPs, which might be related the increased size of the introns; the average intron size of sorghum is 444 bp, but for *Arabidopsis* it is 168 bp [15]. A large number of SNPs was identified to alter in 202 splicing sites, 453 gain of stop codons, and 125 loss of stop codons. These alterations could lead to open reading frames extension, functional gene expression failure, or intron size increase [8, 11, 33]. Besides, the proportion of 3 bp Indels was observed to be the highest in coding regions. This might be due to the loss or increase of three bases results in the deletion or addition of a single amino acid without disrupting the overall reading frame [34], which could be a protection means to avoid the drastic changes of the genetic coding information, and then reduce damage to organisms due to natural variation. In addition, Indels with no multiples of 3 bp were rare in coding regions but relatively common in non-coding regions, because most of frameshift mutations is harmful to sorghum survival [15]. Compared to the BTx623 reference genome, a large number of SVs and CNVs was presented in the Hongyingzi genome, and the annotations of SVs and CNVs were similar to that of SNPs and Indels.

Compared to the BTx623 reference genome, there were 29,614 gene variations in the Hongyingzi genome and Indels accounted for most of the gene variations. However, previous studies reported that SNPs accounted for most of the gene variations in *Arabidopsis* [35] and sorghum [15]. There are two possible reasons: 1) different materials used in different research, 2) limitations of early sequencing technology. Studies of SVs and CNVs in sorghum lag behind those in other plants. Recent studies in maize showed it potentially contributed to the heterosis during domestication and disease responses [36, 37]. Thus, we should focused on non-synonymous SNPs and Indels in coding regions for subsequent analysis of mutative genes. In our study, GO annotation showed that the mutative genes were equal distribution in different GO term. This indicates that SNPs and Indels may share similar survival and distribution patterns, although the origins and scales may different for affected genome segments.

Tannin, also known as condensed tannin or proanthocyanidins, is oligomers and polymers of flavan-3-ols [16, 17]. Sorghum has been the raw material for making famous liquor because of its grains containing tannin, and contributed special taste to Moutai-flavor liquor [18, 19]. Compared to the BTx623 reference genome, 35 gene variations were related to the tannin synthesis in the Hongyingzi genome. The genes involved in the MATE transporter, CHS, AHA10 transporter, DFR, LAC15, F3′H, F3H, OMT, F3′5′H, SGT, FLS, and CHI. Of these 35 gene variations, 3 MATE, 6 CHS, 2 AHA10, 2 DFR, 1 LAC15, 1 F3H, 2 F3′H, 2 OMT, 1 F3′5′H, 1 FLS and 1 CHI had only non-synonymous SNP variation, 2 MATE and 1 AHA10 had only non-frameshift insertion variation, 2 DFR had only non-frameshift deletion variation, 1 MATE had both non-synonymous SNP and non-frameshift insertion variations, 1 MATE, 1 CHS and 1 F3′H had both non-synonymous SNP and non-frameshift deletion variations, 1 AHA10 had both non-synonymous SNP, frameshift insertionand, non-frameshift deletion, non-frameshift insertion and gain of stop codons variations, 1 LAC15 had both non-synonymous SNP and frameshift deletion variations, and 1 LAC15 had both non-synonymous SNP, non-frameshift deletion, and frameshift insertion variations. There were no other reports about these variation and whether those genes only presence in Hongyingzi should for further studies. Furtherly, we selected 11 genes with only 1 bp non-synonymous SNP variation in coding regions to DNA sequence alignment, and found that *Sobic.001G012600* had an alteration from arginine to serine, *Sobic.001G543900* had an alteration from proline to alanine, *Sobic.003G230900* had an alteration from proline to serine, *Sobic.004G179000* had an alteration from arginine to cysteine, *Sobic.004G310100* had an alteration from glycine to serine, *Sobic.005G136200* had an alteration from alanine to glycine, *Sobic.005G136300* had an alteration from alanine to threonine, *Sobic.007G165500* had an alteration from proline to glutamine, *Sobic.008G030100* had an alteration from threonine to arginine, *Sobic.010G207800* had an alteration from isoleucine to threonine, and *Sobic.010G052200* had an alteration from alanine to glycine. Whether those alterations affect the tannin synthesis in Hongyingzi should for further studies. Expectantly, these variations would provide theoretical supports for the molecular markers developments and gene cloning, and the genetic improvement of liquor-making sorghum based on the genome editing technology.

## Conclusions

This is the first report of genome-wide variations analysis in liquor-making sorghum. High-density SNP, Indel, SV, and CNV reported here will be a valuable resource for future gene-phenotype studies and the molecular breeding of liquor-making sorghum. Gene variations involved in tannin synthesis reported here will provide theoretical basis for marker developing and gene cloning.

## Supplementary information

**Additional file 1: Figure S1** and **Figure S2.**

**Additional file 2: Figure S3** and **Figure S4.**

**Additional file 3: Figure S5** and **Figure S6.**

## Data Availability

The raw sequence data in the fastq format from this study were deposited in the NCBI Short Read Archive (SRA) under the accession number SRR11355755. All data produced by the study are disclosed in the manuscript and the additional files.

## Abbreviations

AHA10: ATPase isoform 10
CHI: Chalcone isomerase
CHS: Chalcone synthase
CNV: Copy number variation
DFR: Dihydroflavonol-4-reductase
F3H: Flavanone 3-hydroxylase
F3′H: Flavonol 3′-hydroxylase
F3′5′H: Flavonoid 3′5′ hydroxylase
FLS: Flavonol synthase
GO: Gene Ontology
Indel: Small fragments insertion and deletion
KEGG: Kyoto Encyclopedia of Genes and Genomes
LAC15: Laccase 15
MATE: Multidrug and toxic efflux
OMT: *O*-methyltransferase
SNP: Single nucleotide polymorphism
SV: Structural variation
SGT: UDP-glucose:sterol-glucosyltransferase
