# Supplementary figures and images for: Genome-wide variations analysis of sorghum cultivar Hongyingzi for brewing Moutai liquor
Source: Hereditas. 2020 May 14;157:19. doi: 10.1186/s41065-020-00130-4 (PMC7227080; doi:10.1186/s41065-020-00130-4)

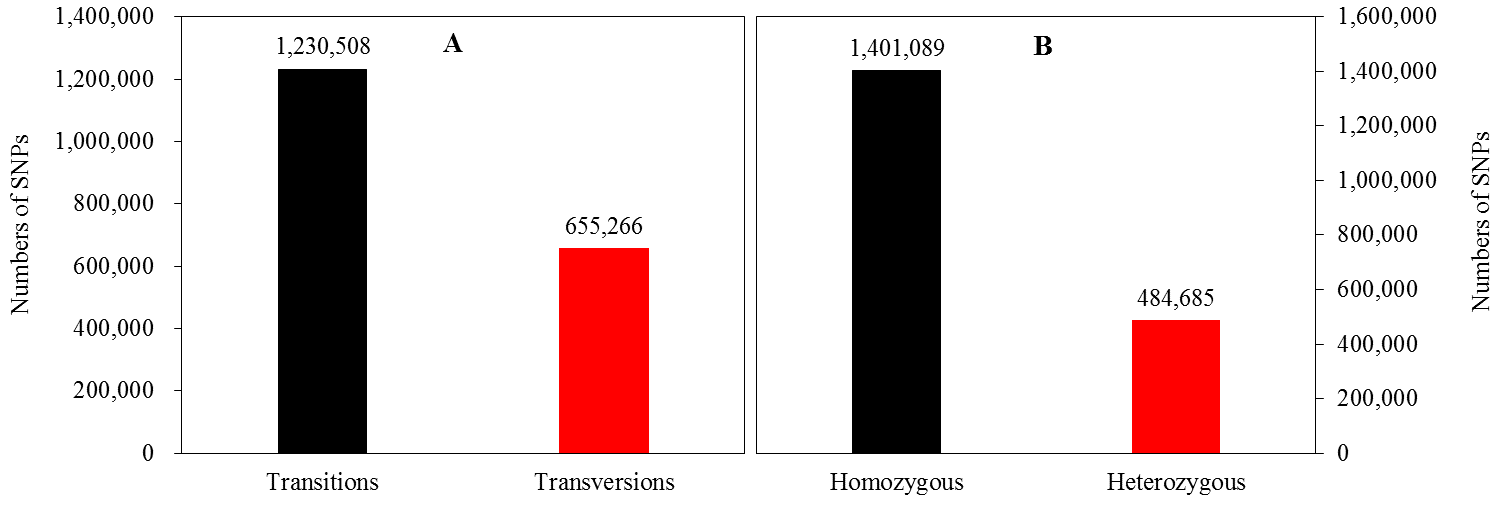

Supplement: Supplementary file 1 — Additional file 1: Figure S1 and Figure S2. [file 41065_2020_130_MOESM1_ESM.zip › Fig.S1_ESM.png]

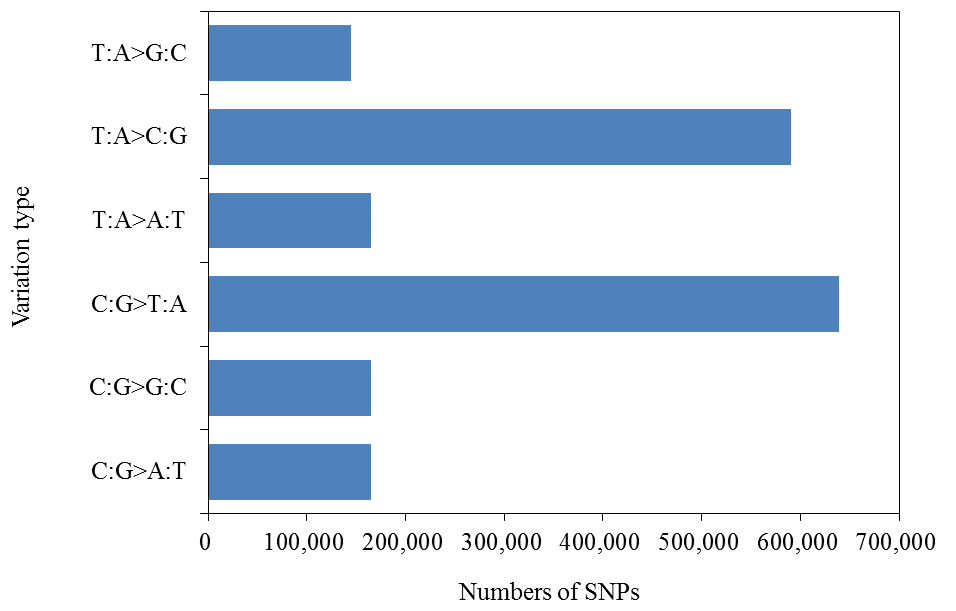

Supplement: Supplementary file 1 — Additional file 1: Figure S1 and Figure S2. [file 41065_2020_130_MOESM1_ESM.zip › Fig.S2_ESM.png]

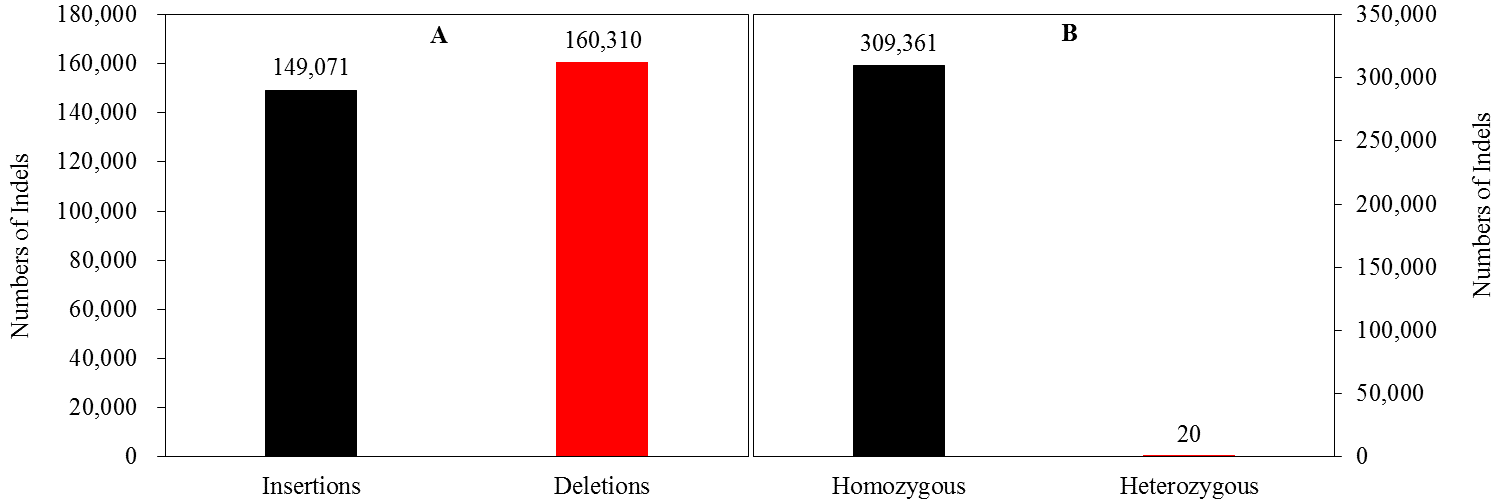

Supplement: Supplementary file 2 — Additional file 2: Figure S3 and Figure S4. [file 41065_2020_130_MOESM2_ESM.zip › Fig.S3_ESM.png]

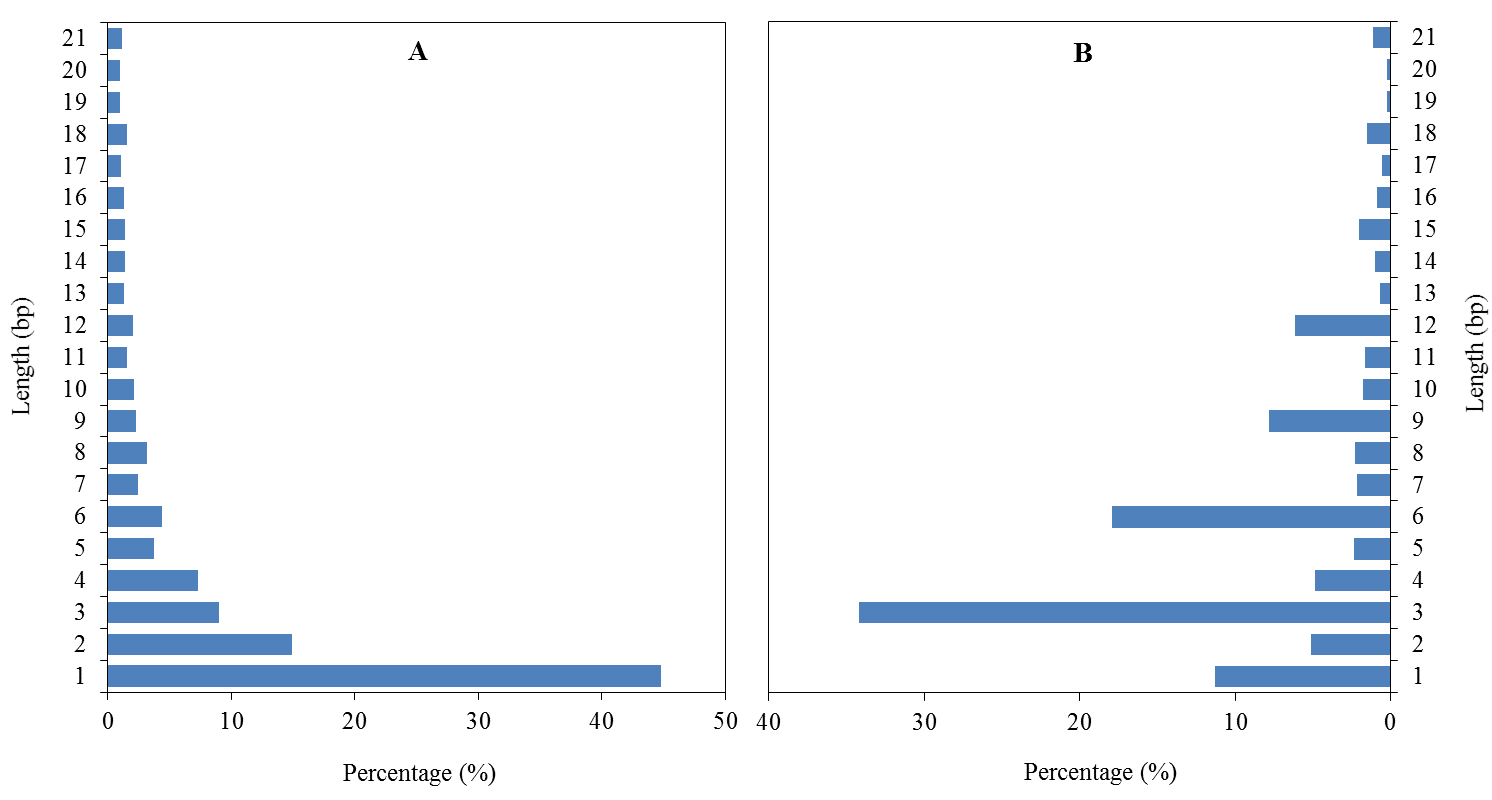

Supplement: Supplementary file 2 — Additional file 2: Figure S3 and Figure S4. [file 41065_2020_130_MOESM2_ESM.zip › Fig.S4_ESM.png]

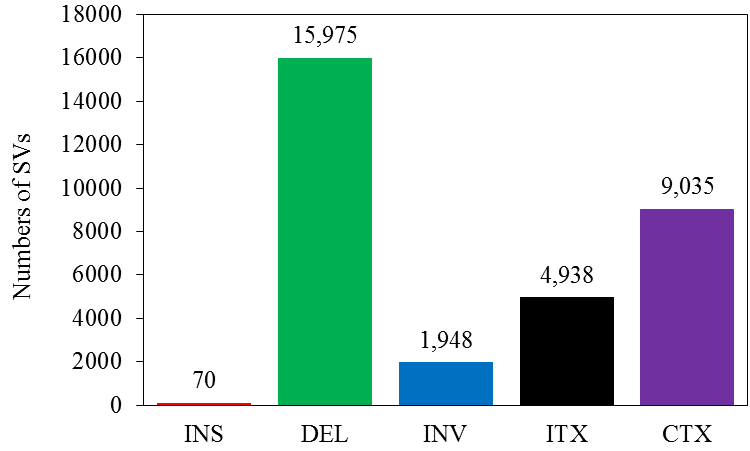

Supplement: Supplementary file 3 — Additional file 3: Figure S5 and Figure S6. [file 41065_2020_130_MOESM3_ESM.zip › Fig.S5_ESM.png]

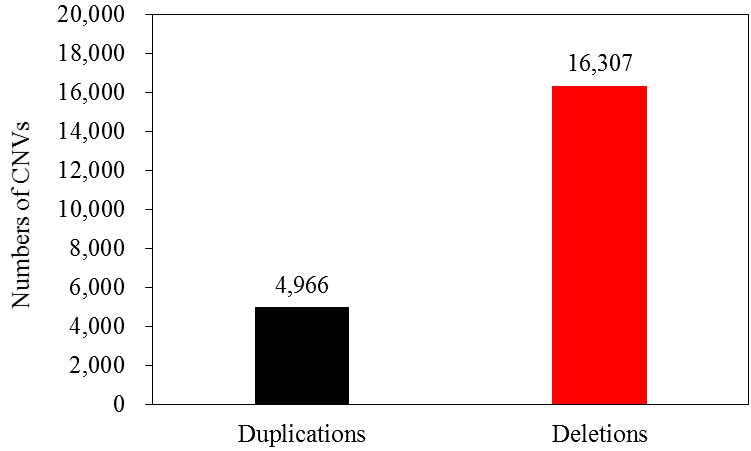

Supplement: Supplementary file 3 — Additional file 3: Figure S5 and Figure S6. [file 41065_2020_130_MOESM3_ESM.zip › Fig.S6_ESM.png]
